# Exploring the role of GHRH antagonist MIA-602 in overcoming Doxorubicin-resistance in acute myeloid leukemia

**DOI:** 10.18632/oncotarget.28579

**Published:** 2024-04-08

**Authors:** Simonetta I. Gaumond, Rama Abdin, Joel Costoya, Andrew V. Schally, Joaquin J. Jimenez

**Affiliations:** ^1^Dr. Philip Frost Department of Dermatology and Cutaneous Surgery, Miller School of Medicine, University of Miami, Miami, FL 33136, USA; ^2^Charles E. Schmidt College of Medicine, Florida Atlantic University, Boca Raton, FL 33431, USA; ^3^Veterans Affairs Medical Center, Miami, FL 33125, USA

**Keywords:** leukemia, AML, resistance, growth hormone-releasing hormone, MIA-602

## Abstract

Acute myeloid leukemia (AML) is characterized by the rapid proliferation of mutagenic hematopoietic progenitors in the bone marrow. Conventional therapies include chemotherapy and bone marrow stem cell transplantation; however, they are often associated with poor prognosis. Notably, growth hormone-releasing hormone (GHRH) receptor antagonist MIA-602 has been shown to impede the growth of various human cancer cell lines, including AML. This investigation examined the impact of MIA-602 as monotherapy and in combination with Doxorubicin on three Doxorubicin-resistant AML cell lines, KG-1A, U-937, and K-562. The *in vitro* results revealed a significant reduction in cell viability for all treated wild-type cells. Doxorubicin-resistant clones were similarly susceptible to MIA-602 as the wild-type counterpart. Our *in vivo* experiment of xenografted nude mice with Doxorubicin-resistant K-562 revealed a reduction in tumor volume with MIA-602 treatment compared to control. Our study demonstrates that these three AML cell lines, and their Doxorubicin-resistant clones, are susceptible to GHRH antagonist MIA-602.

## INTRODUCTION

Acute myeloid leukemia (AML) is a cancer of hematopoietic origin defined by clonal cytogenic expansion of abnormally or poorly differentiated cells [1, 2]. Two recent classification systems have been published to reflect advancements in the genetic, pathologic, and clinical understanding of the disease. Both the World Health Organization (WHO) [3] and International Consensus Classification (ICC) [4] systems expand upon the genetic derangements of AML subtypes, and while these classifications systems do differ on certain qualifying mutations or blast thresholds to characterize certain types of AML, their overall goal is to decrease confusion caused by overlap between AML categories. The emergence of these new classification systems highlights key breakthroughs and developments within the field which continues to evolve our understanding of the disease with the ultimate goal of streamlining diagnosis and treatment. The first step in formulating a treatment plan for newly diagnosed AML involves establishing whether a patient is fit for intensive chemotherapy [1, 2]. The typical treatment paradigm of newly diagnosed AML includes intensive chemotherapy to achieve complete remission followed by post-remission therapy such as chemotherapy and/or stem cell transplantation [1]. There are multiple approved therapies for the treatment of AML, and treatment choice depends on various patient factors [5]. The gold standard of induction therapy in eligible patients is anthracycline and cytarabine therapy [1, 2]. In patients 60 years of age or younger, a complete response can be seen in 60% to 85% of adults, however, there is a high risk of relapse typically within 3 years after diagnosis [2]. Negative prognostic factors associated with relapse include short duration of remission, genetic derangements, previous allogeneic transplantation, older age, and concomitant comorbidities [2]. Continued investigation of therapeutic agents is thus imperative given the potential for relapse despite treatment. However, drug resistance can be a prominent barrier to treatment success [6]. Drug resistance can be divided into two main categories: primary drug resistance and acquired drug resistance. The underlying mechanisms of these pathways involve drug resistance-related proteins and enzymes, genes, microRNAs, and aberrant signaling pathways [6].

Growth hormone-releasing hormone (GHRH) is a neuropeptide hormone released from the hypothalamus. Canonically, it binds pituitary GHRH receptor (GHRH-R) to induce the release of growth hormone. However, its effects are not limited to this endocrine axis; in fact, it has been found to act as a growth factor in many cancer types as well as normal tissue in an autocrine/paracrine fashion [7, 8]. We have previously demonstrated the expression of GHRH-R in human AML cell lines K-562, THP-1, and KG-1A [9]. We have also demonstrated the ability of MIA-602, a GHRH antagonist, to inhibit the proliferation of these leukemic cells *in vitro* as well as in preclinical mouse models [9, 10]. Furthermore, we have investigated MIA-602’s utility in treating all-trans retinoic acid (ATRA) and arsenic trioxide (ATO) resistant cells [10]. Given the role of GHRH in multiple cancer types, it is possible that GHRH antagonists may offer an alternative treatment approach for AML as well as drug-resistant AML, which may circumvent the side effects associated with standard chemotherapy. Established cell lines have become a model of investigating drug resistance, and the creation of drug resistant cell lines has facilitated the study of newly emerging therapies targeting drug resistance. In this study, we investigate the effects of MIA-602 on three AML cell lines, KG-1A, U-937, and K-562, both *in vitro* and *in vivo*.

## RESULTS

### *In vitro* results

Our preliminary study tested varying concentrations of Doxorubicin ranging from 0.005, 0.01 and 0.05 μg/ml, in which we determined the ED-50 to be 0.01 μg [10]. We then cultured both wild-type (W-T) and Doxorubicin-resistant (D-R) clones with MIA-602 at concentrations ranging from 0.05 μmol/L to 5 μmol/L, for 24 and 48 hours. The optimal concentration of MIA-602 was determined to be 5 μmol/L [9, 10]. We then demonstrated the presence of GHRH receptor (GHRH-R) in both W-T and D-R cell lines by western blot [10]. Since we have previously shown that both the wild-type and the Doxorubicin clones had positive expression of GHRH-R, we hypothesize that MIA-602 could be a beneficial targeted therapy for AML. Our preliminary study also demonstrated that treatment with MIA-602 caused a significant dose- and time-dependent decrease in cell proliferation across all six cell lines [10].

**Figure 1:**
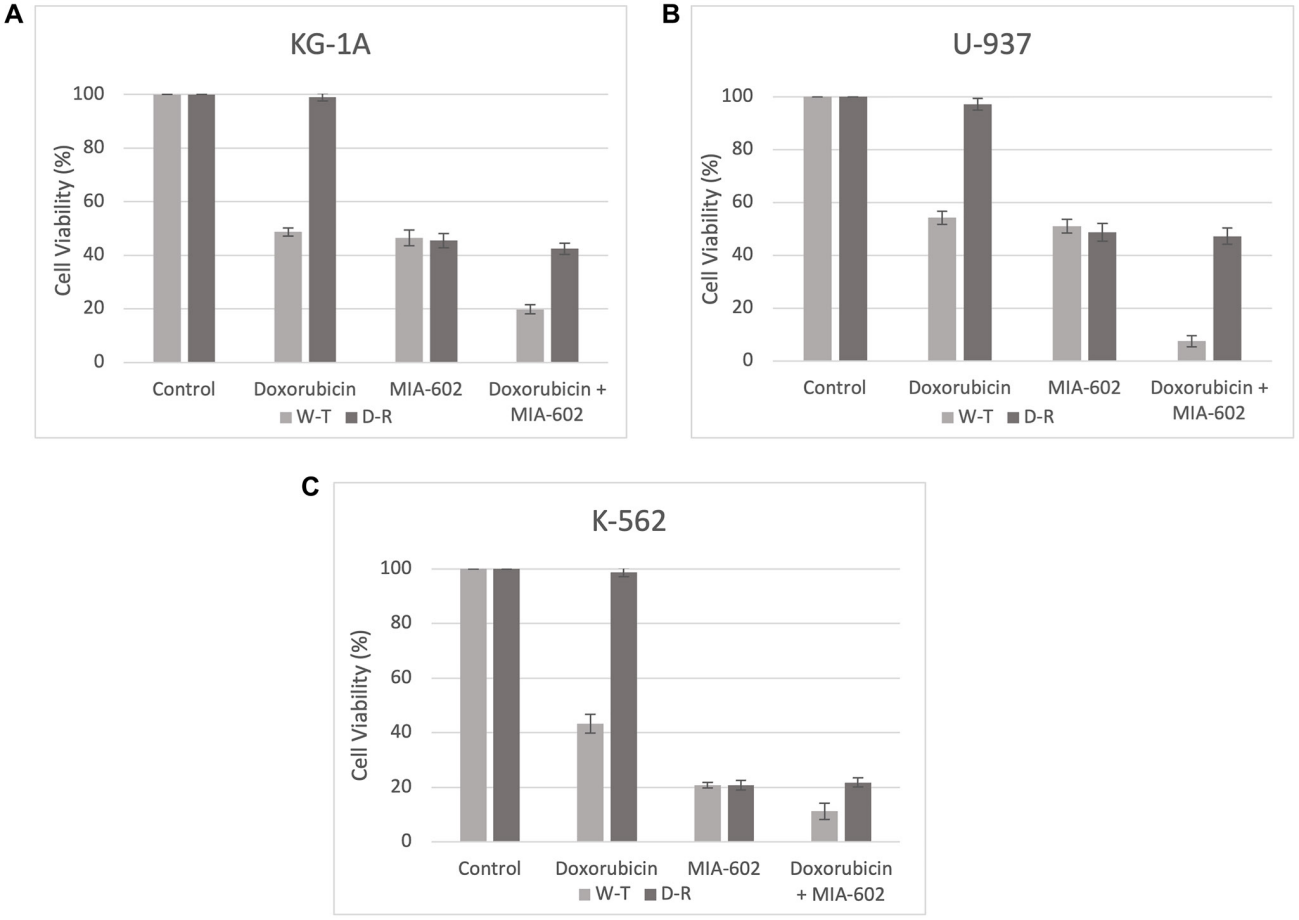
Cell viability (%) after 48 hours across three AML cell lines, KG-1A (**A**), U-937 (**B**), and K-562 (**C**). Wild-type (W-T) and Doxorubicin-Resistant (D-R) clones were treated with either control diluent, Doxorubicin, MIA-602, or a combination of Doxorubicin and MIA-602. Treatment with MIA-602 was effective in reducing cell viability across all cancer cell lines.

To assess the impact of MIA-602 targeted therapy, we cultured three Doxorubicin-resistant AML cell lines (KG-1A, U-937, and K-562) in the presence of Doxorubicin alone, MIA-602 alone, and a combination of Doxorubicin and MIA-602. The wild-type (W-T) and Doxorubicin-resistant (D-R) clones were treated with either control diluent, Doxorubicin (0.01 μg), MIA-602 (5 μmol/L), or a combination of Doxorubicin (0.01 μg) and MIA-602 (5 μmol/L). After 48 hours of incubation, all three Doxorubicin-resistant cancer cell lines demonstrated no reduction in cell viability with Doxorubicin treatment (Figure 1). When treated with Doxorubicin, the W-T KG-1A cells demonstrated, on average, a 51.25% decrease in cell viability, while the U-937 W-T cells decreased by 45.75% and K-562 W-T cells by 56.75% (Figure 1). Treatment with MIA-602 showed a comparable decrease for both W-T and D-R KG-1A clones with a 53.5% and 54.5% decrease in cell viability, respectively (Figure 1A). Similarly, the U-937 cell viability decreased by 49% in the W-T cells and 51.25% in the D-R clones following MIA-602 treatment (Figure 1B). Moreover, cell viability decreased by 79.25% in both the W-T and D-R K-562 clones treated with MIA-602 (Figure 1C). The decrease in viability of Doxorubicin-resistant cell lines after MIA-602 monotherapy emphasizes its distinct mechanism of action from that of Doxorubicin. Combination treatment with Doxorubicin and MIA-602 resulted in an 80.25% decrease in W-T KG-1A cell viability and a 57.5% reduction for their D-R counterpart (Figure 1A). The U-937 cancer cell line responded to combination treatment with a 92.5% decrease in W-T cells and a 52.75% decrease in the D-R resistant strain (Figure 1B). As for the K-562 cells, combination resulted in an 88.75% reduction in W-T cell viability and 78.25% for D-R cells (Figure 1C).

### *In vivo* results

For our animal study, we randomly divided nude mice into two groups of 10 animals each. Both groups were transplanted with Doxorubicin-resistant K-562 cells, with the first group receiving a control diluent and the second receiving MIA-602 treatment. MIA-602 was administered at a dose of 10 μg twice daily for 28 days. Growth of the xenographed Doxorubicin-resistant K-562 tumors was evaluated weekly. After 28 days, treatment with the control diluent resulted in a tumor volume of 1114 mm^3^, while MIA-602 monotherapy resulted in a tumor volume of 629 mm^3^ (Table 1). Notably, treatment with MIA-602 decreased the final tumor volume by 485 mm^3^. This finding further emphasizes the beneficial outcomes of MIA-602 monotherapy for individuals who have developed resistance to Doxorubicin.

**Table 1:** Treatment with MIA-602 on Doxorubicin-resistant K-562 tumors results in decreased weight and volume after 28 days

	Final tumor weight (mg)	Final tumor volume (mm^3^)
**K-562 Control**	1512 ± 88	1114 ± 71
**K-562 Treated**	890 ± 59.5	629 ± 48.3

## DISCUSSION

These results suggest that MIA-602, a GHRH antagonist, may be a viable therapeutic approach to addressing Doxorubicin-resistant AML. We observed a substantial decrease in cell viability in Doxorubicin-resistant KG-1A, U-937, and K-562 cells when exposed to MIA-602 alone compared to Doxorubicin alone (Figure 1). The three cell lines chosen are commonly used *in vitro* models of AML. The variability in the strength of response to MIA-602 and Doxorubicin across cell lines (Figure 1) represents the potential variability in response in human patient populations. We have previously shown the presence of GHRH-R in human AML cell lines K-562, THP-1, and KG-1A as well as the ability of MIA-602 to inhibit their proliferation [9]. The presence of this receptor offers the foundation for a novel approach to the treatment of cancer with a non-cytotoxic agent, MIA-602, given the extensive adverse effects associated with chemotherapy. Furthermore, it has been proposed that while GHRH analogs of the Miami family can inhibit tumor growth, they do not have a strong endocrine inhibitory effect [11]. GHRH antagonists have been implicated in the *in vitro* inhibition of a variety of cancer cell lines including but not limited to APL [10], AML [9], estrogen independent breast cancer [12], clear cell ovarian cancer [12], glioblastoma [12], gastric cancer [13], prostate cancer [11, 14], and endometrial adenocarcinoma [11]. The inhibition of proliferation by MIA-602 is mediated though multiple mechanisms. These include the downregulation of NF-κB and beta-catenin [12], upregulation of caveolin-1 and E-cadherin [12], upregulation of pro-apoptotic pathways such as CAS9 [9], modulation of inflammatory cytokines [15], and the inhibition of Akt [9]. The anti-oncogenic mechanism exerted by MIA-602 likely varies based upon cancer type and even individual cell line qualities, however, it is evident that MIA-602 exerts its anti-cancer effects through the modulation of various factors which promote oncogenesis including but not limited to cellular proliferation, survival, and motility. Our previous work has demonstrated the synergistic effect of combination Doxorubicin and MIA-602 on the K-562 cell line [10]. Interestingly, in this study, the U-937 and KG-1A W-T cell lines demonstrated a more prominent synergistic effect of combination treatment than that of K-562. Across all D-R cell lines, a sustained decrease in cell viability was observed with combination treatment as compared to MIA-602 monotherapy, suggesting that MIA-602 is unaffected by Doxorubicin-resistance, and inflicts its inhibitory effects through a distinct apoptotic pathway than that of Doxorubicin. This may involve the aforementioned mechanisms implicated in other cancer types, and it would be interesting to investigate the specific pathways elicited by MIA-602 in D-R AML cell lines.

**Figure 2:**
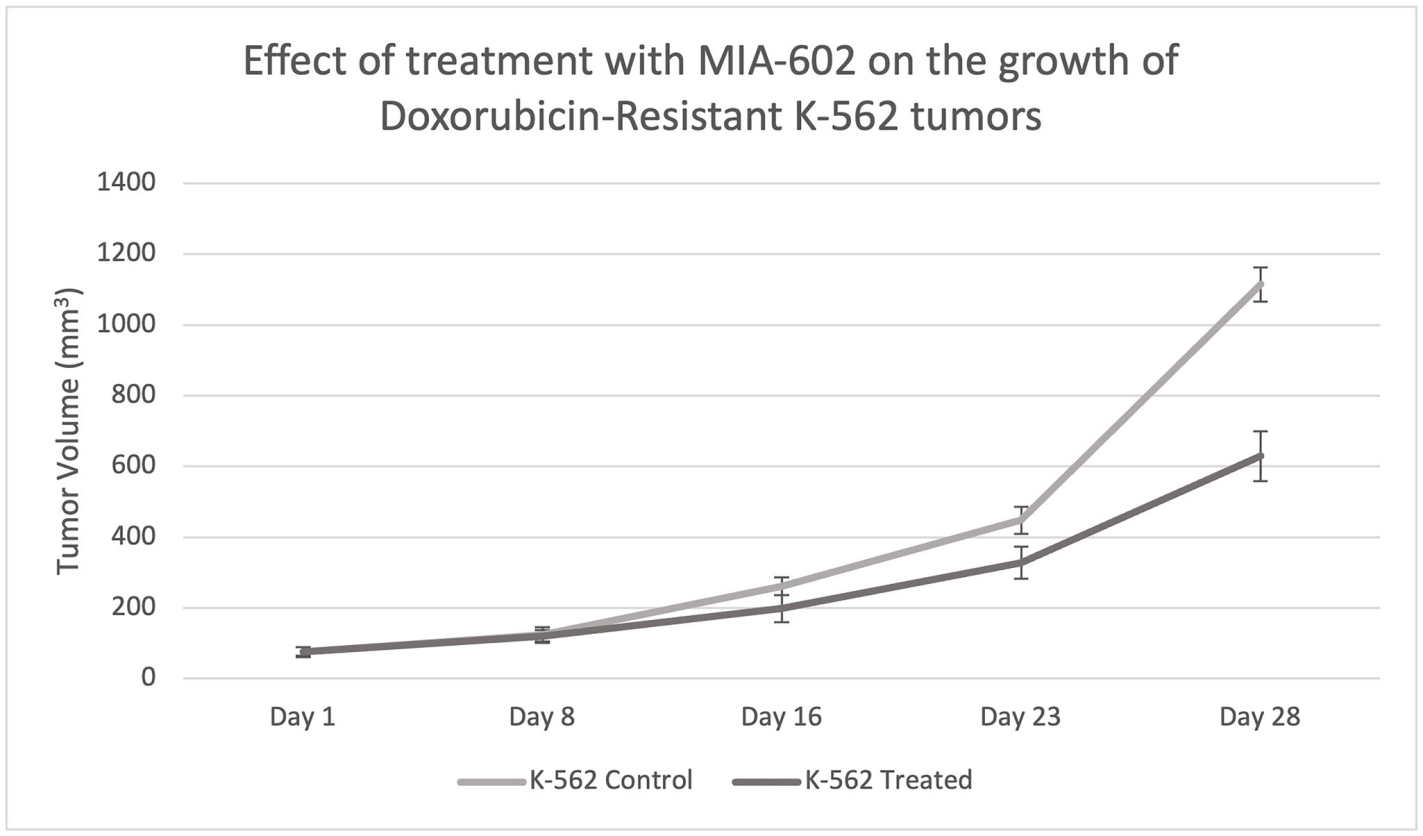
Effect of treatment with MIA-602 on the growth of Doxorubicin-resistant K-562 tumors xenographed into nude mice. After 28 days, treatment with MIA-602 significantly reduced tumor volume compared to the control diluent.

The *in vitro* anti-oncogenic effects of GHRH receptor antagonists have also been corroborated by *in vivo* studies [9, 11, 13–16]. In our study investigating the effects of MIA-602 on HCC-1806 and MX-1 human triple negative breast cancers xenografted into nude mice, MIA-602 treatment at a concentration of 5 μg/day for 5 weeks significantly inhibited mean tumor volume of HCC-1806 tumors and of MX-1 tumors compared to controls [15]. Furthermore, the expression of GHRH and GHRH-R genes was significantly decreased with treatment as compared to controls [15], another plausible mechanism of GHRH antagonist anti-oncogenic action. Further studies are needed to investigate the expression of GHRH receptors in the AML patient population, including differences in expression levels between genetic subtypes of AML, to understand the therapeutic potential of GHRH antagonism [9]. Given these findings, we investigated whether these anti-tumor effects would persist in HCC-1806 and Doxorubicin-resistant MX-1 human TNBC cell lines xenografted into nude mice [16]. Indeed, treatment with MIA-602 significantly reduced the growth of HCC-1806 and Doxorubicin-resistant MX-1 tumors when compared to control. For both tumor types, the growth of tumors treated with the combination MIA-602 and Doxorubicin was significantly smaller than that of controls (*P* < 0.001), and tumors treated with either MIA-602 alone (*P* < 0.05) or Doxorubicin alone (*P* < 0.001) [16]. Furthermore, real-time polymerase chain reaction (RT-PCR) analysis revealed that the expression of genes (MDR1 and NANOG) involved in drug resistance was reduced by treatment with MIA-602 [16]. In this study, treatment of Doxorubicin-resistant K-562 tumors with MIA-602 10 μg twice a day for 28 days decreased tumor growth and volume compared to control (Figure 2). This is consistent with *in vivo* models of treatment-resistant APL in which ATRA/ATO resistant NB4-RAA cell lines were xenografted into nude mice, and MIA-602 10 μg twice a day for 30 days was tested. NB4-RAA treated tumor volumes were significantly reduced, further emphasizing MIA-602’s action through a unique pathway, unaffected by chemotherapy-resistance [10].

In this study we investigated the influence of MIA-602 on Doxorubicin-resistant AML cell lines K-562, KG-1A, and U-937, as well as its effect on Doxorubicin-resistant K-562 tumors xenographed into nude mice. Our prior studies established a foundation of evidence proving the existence of GHRH-R on AML cell lines as well as the ability of MIA-602 to circumvent ATRA and ATO resistance [10]. GHRH antagonists are non-cytotoxic agents which may serve as therapeutic alternatives or adjuvants to cytotoxic chemotherapeutic agents. The anti-oncogenic mechanisms of GHRH antagonists are vast, and likely involve crosstalk between multiple biologic pathways dependent upon cancer type. Future studies may explore the transcriptional effects of GHRH antagonism in Doxorubicin-resistant AML cell lines, and the influence of genetic AML subtypes on these effects. Our results reveal that MIA-602 may be a useful treatment for Doxorubicin-resistant AML, with the potential for enhancing clinical outcomes of AML therapy.

## MATERIALS AND METHODS

### Peptides and reagents

Synthesis of growth hormone-releasing hormone antagonist MIA-602 was obtained by solid phase method and then purified by reverse-phase high performance liquid chromatography (HPLC) as described previously [11]. The chemical composition of MIA-602 is (PhAc-Ada^0^-Tyr^1^, D-Arg^2^, Fpa5^6^, Ala^8^, Har^9^, Tyr (Me)^10^, His^11^, Orn^12^, Abu^15^, His^20^, Orn^21^, Nle^27^, D-Arg^28^, Har^29^) hGH-RH (1-29) NH_2_. The following abbreviations were used to represent non-coded amino acids and acyl groups: Abu (alpha-aminobutyric acid), Ada (12-aminododecanoyl), Fpa5 (pentafluorophenylalanine), Har (homoarginine), Nle (norleucine), Orn (ornithine), PhAc (phenylacetyl), and Tyr (Me) (O-methyl-tyrosine). The peptide was dissolved *in vitro* in dimethyl sulfide (DMSO) and diluted with incubation media, ensuring DMSO concentration never exceeded 0.1%. Conversely, *in vivo*, MIA-602 was dissolved in DMSO and diluted with sterile aqueous 10% phosphate-buffered saline (PBS) pH 7.4 (1X) solution. This served as vehicle control.

### Doxorubicin-resistant cell lines

Cell lines were developed in Dr. Jimenez’s lab from the parent cells line by culturing them in the presence of increasing concentrations of Doxorubicin. This process required multiple subcultures over a period of 12 months.

### Cell culture

Human myeloid leukemia cell lines (KG-1A, U-937, and K-562) were obtained from American Type Culture Collection (ATCC, Manassas, VA, USA). Iscove’s modified Dulbecco’s medium (IMDM) was used to maintain KG-1A and K-562, while U-937 cells were cultured in RPMI-1540 medium. Both media were supplemented with 2 mmol/L L-Glutamine, 25 mmol/L HEPES, 10% FBS, and 50 μg/mL gentamicin. Cells were cultured in an incubator at 5% CO_2_ with 98% humidity at 37°C.

### Cell proliferation

Cells were seeded onto 12-well microplates at a density of 2.5 × 10^5^ cells/mL in their corresponding media supplemented with 1% FBS, 2 mmol/L L-glutamine, 25 mmol/L HEPES, and 50 μg/mL gentamicin. Treatment of the cells with MIA-602 was done at concentrations of 0.05, 0.5 and 5 μmol/L for 24 and 48 h. Each treatment was performed in quadruplicate. Cell proliferation was evaluated by using the MOXI Mini Automated Cell Counter (Orflo Technologies, Ketchum, ID, USA).

### *In vivo* experiment in mice

Female 8-week-old athymic nude mice (Hsd: AthymicNude-Foxn1^nu^) were obtained from Harlan Laboratories (Indianapolis, IN). They were housed in temperature-controlled sterile cages in a room with a 12-h light/12-h dark schedule. Autoclaved chow and water ad libitum was provided for the mice. Xenografts of human myeloid leukemia cells K-562 were introduced by subcutaneous (s.c.) injection of 1 × 10^7^ cells into the right flank of the nude mice. Once tumors reached an appropriate volume (~40 mm^3^), mice were randomly divided into two groups comprised of 10 mice each. Mice assigned to the control group were given s.c. injections of PBS (100 μl) containing 0.1% DMSO, twice a day in their left flank. Mice assigned to the treatment group received s.c. injections of MIA-602 at a dose of 10 μg, also twice a day in their left flank. Measurements of tumor volume (length × width × height × 0.5236) and body weight were obtained every 7 days for a total of 28 days. All animal procedures were performed in compliance with the National Institutes of Health Guide for the Care and Use of Laboratory Animals and approved by the Animal Care and Use Committee of the University of Miami.
